# Inter-limb sensorimotor and functional performance asymmetries in elite wrestlers with unilateral knee injury

**DOI:** 10.3389/fspor.2026.1850922

**Published:** 2026-06-24

**Authors:** İmge Nas, Begüm Büyükerik, Dilan Yetgil

**Affiliations:** 1 Department of Physiotherapy and Rehabilitation, Faculty of Health Sciences, Istanbul Kultur University, Istanbul, Türkiye; 2 Department of Physiotherapy and Rehabilitation, Graduate School of Health Sciences, Istanbul Medipol University, Istanbul, Türkiye; 3 Department of Medical Services and Techniques, Physiotherapy Program, Vocational School, Biruni University, Istanbul, Türkiye; 4 Department of Physical Therapy and Rehabilitation, Medicana Avcılar Hospital, Istanbul, Türkiye

**Keywords:** dynamic postural control, hop test, inter-limb asymmetry, knee injury, proprioception, wrestling

## Abstract

**Introduction:**

Knee injuries are common in wrestling and may result in persistent sensorimotor deficits and inter-limb asymmetries affecting performance. This study compared proprioceptive accuracy, dynamic postural control, and functional performance between injured and uninjured limbs in elite wrestlers with a history of unilateral knee injury.

**Methods:**

Twenty elite wrestlers (10 females, 10 males) who had returned to full participation in training participated in this cross-sectional comparative study. Proprioception was assessed using joint position sense (JPS) at 15°, 30°, and 60° of knee flexion. Dynamic balance and functional performance were evaluated using the Y-Balance Test (YBT) and the single-leg hop test. Pain and subjective knee function were measured using the Visual Analog Scale (VAS), International Knee Documentation Committee (IKDC), and Lysholm scores. Paired comparisons and correlation analyses were performed.

**Results:**

The injured limb showed significantly greater JPS error at 15° (*p* = 0.001, dz = 0.89) and reduced performance in the posteromedial direction of the YBT (*p* = 0.005, dz = 0.71). Hop test distance was also significantly lower in the injured limb (*p* = 0.001; dz = 0.92). Moderate correlations were observed between hop performance, IKDC scores, pain intensity, and YBT reach distances.

**Discussion:**

Elite wrestlers may exhibit persistent sensorimotor and functional performance asymmetries despite returning to sport. These findings suggest that return to sport may not reflect full neuromuscular recovery. Comprehensive assessment of sensorimotor function and inter-limb performance asymmetry may help identify residual impairments and improve return-to-sport decision-making.

## 1 Introduction

Wrestling is a high-intensity combat sport requiring rapid changes in body position, explosive lower-extremity actions, and sustained sensorimotor control during dynamic contact maneuvers. During wrestling competitions, athletes must effectively utilize multiple performance components such as balance, agility, explosive power, maximal strength, endurance, coordination, and psychological resilience to achieve technical superiority or to bring their opponent's shoulders to the mat (1, 2). The high-intensity and contact-based nature of wrestling places substantial mechanical stress on the lower extremities and requires efficient neuromuscular control to maintain performance and reduce injury risk (3).

Recent evidence suggests that the incidence of injuries in wrestling has increased over time (4). Lower extremity injuries constitute a substantial proportion of all injuries sustained in wrestling, with epidemiological studies reporting that approximately 30%–40% of injuries in collegiate wrestlers involve the lower extremities. Among these, the knee is one of the most frequently injured anatomical sites (5, 6). This vulnerability is likely related to the sport-specific mechanical demands of wrestling, including repetitive mat contact, rapid changes in direction, and high torsional loads during grappling maneuvers, which expose the knee joint to substantial varus–valgus, rotational, and compressive stresses (7–9). Evidence further suggests that previous injury and repeated exposure to high mechanical loads during sport-specific movements may influence both the incidence of knee injuries and recovery outcomes in wrestlers (10, 11).

Beyond the immediate structural damage, knee injuries may lead to persistent deficits in proprioception, dynamic balance, and functional performance even after athletes return to sport. Importantly, return to sport does not necessarily indicate full neuromuscular recovery, and residual inter-limb asymmetries may persist despite clinical clearance (12). Proprioception plays a key role in joint position awareness and movement coordination, contributing to joint stability during sport-specific actions (13). Similarly, dynamic balance is essential for postural control during rapid directional changes, deceleration, and single-leg loading tasks that are frequently encountered in wrestling (14). Persistent asymmetries in these functions may negatively affect athletic performance and compromise safe return to competition (15, 16).

Despite the high prevalence of knee injuries in wrestling, studies examining the residual functional and sensorimotor deficits in wrestlers who have returned to sport remain limited. Furthermore, few studies have simultaneously evaluated proprioception, dynamic balance, and functional performance when comparing injured and uninjured limbs in elite wrestlers. It remains unclear whether limb-specific sensorimotor asymmetries persist after returning to sport in elite wrestlers. Therefore, the aim of this study was to compare pain intensity, joint position sense, dynamic balance, and lower extremity functional performance between the injured and uninjured limbs in elite wrestlers who had previously sustained a unilateral knee injury and returned to sport, and to identify residual inter-limb asymmetries.

## 2 Materials and methods

### 2.1 Study design

This cross-sectional comparative study evaluated differences in joint position sense, dynamic balance, and lower extremity performance between the injured and uninjured knees of elite wrestlers who had previously sustained a knee injury and returned to sport. Ethical approval for the study was obtained from the Ethics Committee of Istanbul Kultur University (Decision No: 2023/79). The study was conducted in accordance with the Declaration of Helsinki (17). All participants provided written informed consent prior to participation in the study.

### 2.2 Participants

The study was conducted with elite national team wrestlers. A total of 20 elite wrestlers (10 females and 10 males) who had previously sustained a unilateral knee injury and returned to full training participated in the study. At the time of data collection, all athletes were participating in intensive national team training camps during the preseason period. The inclusion criteria for the study were as follows: being an active elite-level wrestler, having a history of unilateral knee injury and subsequent return to sport, and having sustained the injury within the past 3 to 6 months. Athletes who had functional impairments due to neurological or systemic diseases, those with other orthopedic injuries affecting the lower extremities, or those with any health condition that could interfere with the assessment procedures were excluded from the study. Demographic characteristics of the participants, including age, height, body weight, body mass index, and injury-related variables, were recorded.

### 2.3 Outcome measures

All measurements were performed by the same examiner using standardized assessment procedures and established outcome measures at the athletes’ club facilities. Pain intensity, knee function, proprioception, dynamic balance, and functional performance were assessed in a standardized order.

Pain intensity was assessed using the Visual Analog Scale (VAS). The VAS consists of a 10-cm horizontal line, where 0 represents “no pain” and 10 represents “worst imaginable pain.” Participants were asked to indicate their current level of knee pain at the time of assessment. VAS is a valid and reliable tool for assessing pain in individuals with musculoskeletal conditions (18).

Knee function was evaluated using the Lysholm Knee Scoring Scale and the Inter-na-tional Knee Documentation Committee Subjective Knee Form (IKDC). The Lysholm scale consists of eight items assessing symptoms and functional limitations related to knee ligament injuries, with scores ranging from 0 to 100, where higher scores indicate better function (19). The IKDC Subjective Knee Form evaluates symptoms, daily activities, and sports function, with scores expressed as a percentage of the maximum possible score, where higher values indicate better knee function (20).

Proprioception was assessed using the joint position sense (JPS) test with a standard universal goniometer. During the assessment, participants were positioned in a seated posture at the edge of the examination table with their eyes closed. To assess knee joint position sense, the passive-to-active joint repositioning method was used. The examiner passively moved the knee to target angles of 15°, 30°, and 60° of flexion and maintained each position for 5 s. Participants were instructed to memorize the target position before the limb was returned to the starting position. They were then asked to actively reproduce the target angle, and the absolute error between the target and reproduced angles was recorded in degrees. Three trials were performed for each angle, and the mean error was used for analysis. Joint position sense is a valid method for assessing proprioceptive function in individuals with knee conditions (21).

Dynamic balance was assessed using the Y-Balance Test (YBT). Participants were instructed to maintain balance on a single leg while reaching as far as possible in the anterior, posteromedial, and posterolateral directions. Reach distances (in centimeters) were measured and normalized to lower extremity length (22).

Functional performance was assessed using the Single-Leg Hop Test which has demonstrated excellent test–retest reliability (ICC = 0.94) (23). Participants were instructed to start from a standardized position and perform a maximal forward hop, landing on the same limb and maintaining balance for at least 2 s. The hop distance was measured from the starting line to the heel of the landing foot. Trials were considered invalid if participants lost balance or touched the ground with the contralateral limb. Participants performed three successful trials for each limb, and the average hop distance (in centimeters) was used for analysis. Limb symmetry index (LSI) was calculated as (injured limb/uninjured limb) × 100. LSI values were used to describe inter-limb symmetry, with values of ≥90% considered indicative of acceptable symmetry (24, 25).

### 2.4 Statistical analysis

All statistical analyses were performed using IBM SPSS Statistics version 26.0 (IBM Corp., Armonk, NY, USA). The normality of continuous variables was assessed using the Shapiro–Wilk test. Descriptive statistics were presented as mean ± standard deviation (SD) for continuous variables and frequency (percentage) for categorical variables. Comparisons between injured and uninjured limbs were conducted using paired samples t-tests. Bonferroni correction was applied for multiple paired comparisons. Pearson correlation analysis was performed to examine the relationships between clinical symptoms, proprioception, dynamic balance, and functional performance variables after confirming normality assumptions. 95% confidence intervals for correlation coefficients were calculated. Effect sizes for paired comparisons were calculated using Cohen's dz and interpreted as small (0.2), medium (0.5), and large (0.8) (26). Statistical significance was set at *p* < 0.05.

## 3 Results

Twenty elite wrestlers (10 females and 10 males) with a mean age of 23.6 ± 2.2 years participated in the study. The painful knee corresponded to the dominant limb in 9 athletes (45%), while 11 athletes (55%) reported pain in the non-dominant limb. The mean IKDC score was 66.10 ± 8.25 and the Lysholm score was 74.20 ± 13.65, indicating moderate functional limitation, and the average limb symmetry index was 90.71 ± 10.98. The most common injury was anterior cruciate ligament (ACL) injury (60%) (Table 1).

**Table 1:** Demographic and clinical characteristics of the participants.

Characteristics	Mean ± SD
Age (year)	23.65 **±** 2.20
Height (cm)	168.15 ± 8.14
Weight (kg)	63.85 ± 12.03
Body mass index (kg/m^2^)	22.41 ± 2.72
Years in sport	10.20 ± 1.96
Time since injury (months)	5.40 ± 0.88
Number of injuries	1.65 ± 0.58
Time away from sport (weeks)	8.40 ± 4.22
Pain intensity (VAS)	4.75 ± 1.77
Lysholm Knee Score	74.20 ± 13.65
IKDC Total Score	66.10 ± 8.25
Symptoms	21.60 ± 1.90
Sports Activities	29.60 ± 6.89
Function	14.90 ± 2.49
LSI (%)	90.71 ± 10.98

*Variable*	*n (%)*
Sex	
Female	10 (50)
Male	10 (50)
Wrestling style	
Freestyle	16 (80)
Greco-Roman	4 (20)
Dominant limb	
Right	18 (90.0)
Left	2 (10.0)
Injured limb	
Right	9 (45.0)
Left	11 (55.0)
Type of injury	
ACL injury	12 (60.0)
Meniscus (lateral or medial)	5 (25.0)
LCL or MCL injury	3 (15.0)

Mean ± SD, mean ± standard deviation; VAS, visual analog scale; IKDC, International Knee Documentation Committee; LSI, limb symmetry index; ACL, anterior cruciate ligament; LCL, lateral collateral ligament; MCL, medial collateral ligament.

**Figure 1:**
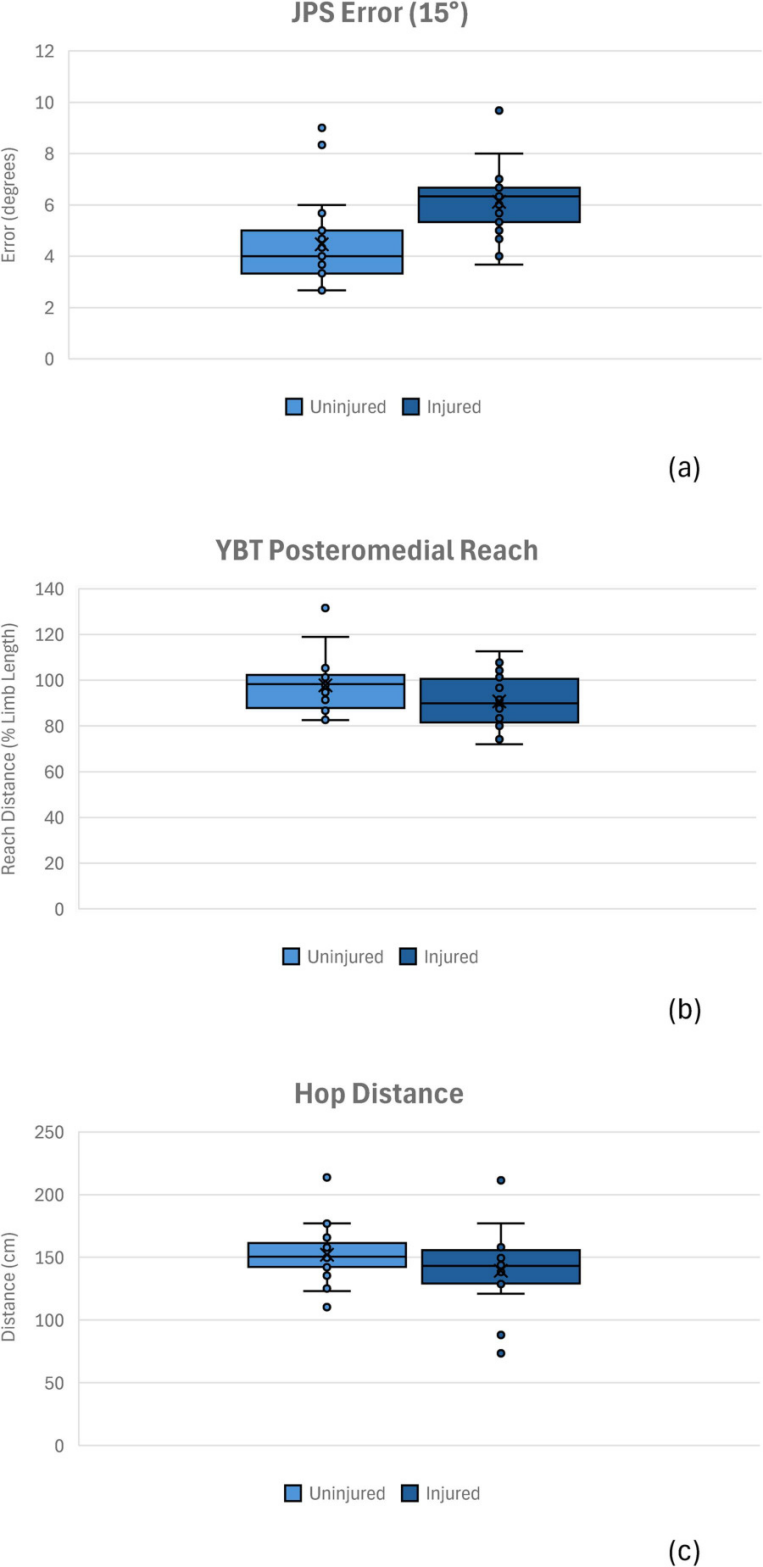
Comparison of injured and uninjured limbs **(a)** joint position sense error at 15° knee flexion; **(b)** posteromedial reach distance in the Y-balance test (% limb length); **(c)** single-leg hop distance (cm).

Paired comparisons revealed a significantly greater JPS error at 15° in injured knees (6.11 ± 1.33°) compared with uninjured knees (4.46 ± 1.73°, *p* = 0.001), with a large effect size (dz = 0.89). No statistically significant differences were observed at 30° or 60° knee flexion angles, although mean errors remained slightly higher in the injured limbs. Dynamic balance analysis showed a significant deficit in the posteromedial reach direction of the YBT in injured limbs (90.66 ± 11.61) compared with uninjured limbs (97.70 ± 12.05; *p* = 0.005; dz = 0.71). No significant differences were observed in the anterior, posterolateral, or composite YBT scores. Functional performance assessed by the single leg hop test revealed significantly shorter jump distances in the injured limb (139.13 ± 30.88 cm) compared with the uninjured limb (152.05 ± 21.94 cm; *p* = 0.001), with a large effect size (dz = 0.92) (Table 2; Figure 1).

**Table 2:** Comparison of proprioception, dynamic balance, and functional performance between injured and uninjured limbs.

Outcome Measure	Injured Limb (*n* = 20) Mean ± SD	Uninjured Limb (*n* = 20) Mean ± SD	*p*-value	Effect Size (dz)	Mean Difference (95% CI)
JPS at 15°	6.11 ± 1.33	4.46 ± 1.73	0.001*	0.89	1.65 (0.78–2.51)
JPS at 30°	4.06 ± 2.50	3.13 ± 1.39	0.075	0.42	0.93 (−0.10–1.97)
JPS at 60°	3.38 ± 1.58	2.65 ± 1.41	0.129	0.35	0.73 (−0.23–1.70)
YBT anterior	80.29 ± 11.98	80.74 ± 10.66	0.821	0.05	−0.45 (−4.60–3.69)
YBT posteromedial	90.66 ± 11.61	97.70 ± 12.05	0.005*	0.71	−7.03 (−11.65– −2.41)
YBT posterolateral	92.21 ± 15.37	93.12 ± 15.25	0.777	0.06	−0.90 (−7.50–5.69)
YBT composite score	87.72 ± 10.84	90.52 ± 9.82	0.083	0.41	−2.79 (−5.99–0.39)
Hop test	139.13 ± 30.88	152.05 ± 21.94	0.001*	0.92	−12.91 (−19.47– −6.36)

Mean ± SD, mean ± standard deviation.; CI, confidence interval; JPS, joint position sense; YBT, Y-balance test.

* *p* < 0.05 indicates statistical significance.

Correlation analysis demonstrated a positive association between IKDC scores and hop test distance (r = 0.538, *p* = 0.014). Pain intensity (VAS) was negatively correlated with hop test distance (r = −0.587, *p* = 0.007). Hop test distance also showed moderate positive correlations with YBT reach distances in the posteromedial (r = 0.561, *p* = 0.010) and posterolateral directions (r = 0.592, *p* = 0.006), as well as with the composite YBT score (r = 0.574, *p* = 0.008). No significant correlations were found between JPS error at 15° and the other clinical or functional variables (Table 3).

**Table 3:** Correlations between clinical, proprioceptive, balance, and functional performance variables.

Variables	r	*p*-value	95% CI
Hop Test vs. IKDC Total Score	0.538	0.014*	0.13–0.79
Hop Test vs. VAS	−0.587	0.007*	−0.82–0.20
Hop Test vs. YBT anterior	0.255	0.278	−0.21–0.63
Hop Test vs. YBT posteromedial	0.561	0.010*	0.16–0.80
Hop Test vs. YBT posterolateral	0.592	0.006*	0.20–0.82
Hop Test vs. YBT composite score	0.574	0.008*	0.18–0.81
JPS 15° Error vs. VAS	−0.017	0.944	−0.46–0.43
JPS 15° Error vs. YBT composite score	0.008	0.972	−0.44–0.45
JPS 15° Error vs. Hop Test	−0.010	0.968	−0.45–0.43

r, Pearson correlation coefficient; CI, confidence interval; VAS, visual analog scale; JPS, joint position sense; YBT, Y-balance test; IKDC, International Knee Documentation Committee.

*p < 0.05 indicates statistical significance.

## 4 Discussion

The present study aimed to compare proprioception, dynamic balance, and functional performance between injured and uninjured limbs in elite wrestlers with a history of unilateral knee injury. The main findings indicated that injured knees demonstrated significantly greater joint position sense error at 15°of knee flexion, reduced reach distance in the posteromedial direction of the Y-Balance Test, and shorter jump distances in the single-leg hop test. In contrast, no significant differences were observed in JPS at higher flexion angles or in most dynamic balance directions. These findings suggest that elite wrestlers with a previous knee injury may demonstrate residual differences in proprioceptive accuracy and functional performance after returning to full training, while balance impairments appear to be direction-specific rather than global.

In addition to these limb-specific differences, the present findings demonstrated that, despite returning to sport, athletes exhibited only moderate functional recovery, as reflected by IKDC and Lysholm scores. Although the mean limb symmetry index approached the commonly accepted threshold of 90%, the considerable variability observed among participants suggests that a substantial proportion of athletes may still present with clinically relevant inter-limb asymmetries. These results indicate that achieving near-threshold symmetrical values may not necessarily reflect complete sensorimotor recovery. Indeed, previous research has shown that commonly used return-to-sport criteria, such as limb symmetry indexes, may overestimate knee function and fail to detect persistent functional deficits following anterior cruciate ligament injury (27). Instead, residual functional differences may remain evident during high-intensity, sport-specific activities, potentially influencing movement control and sport performance.

The greater joint position sense error observed at 15° of knee flexion in the injured limb suggests a persistent deficit in proprioceptive acuity following knee injury. Proprioception is largely mediated by mechanoreceptors located in ligaments, joint capsules, and surrounding musculature, which provide afferent feedback necessary for joint position awareness and sensorimotor control (28). Previous studies have shown that knee injuries, particularly ligamentous injuries, can impair the function of these mechanoreceptors and alter afferent signaling, leading to reduced proprioceptive accuracy even after return to sport (29). Lower knee flexion angles may be particularly sensitive to such deficits because joint stability in near-extension positions relies heavily on proprioceptive feed-back and active neuromuscular control rather than purely mechanical constraints (30). This may explain why the difference in JPS error was more evident at 15° compared with higher flexion angles in the present study. In addition, the inclusion of different knee injury types may have influenced proprioceptive recovery patterns, as different injured structures may affect mechanoreceptor function to varying degrees. Although no statistically significant differences were observed at 30° and 60° knee flexion angles, the observed effect sizes may still indicate subtle sensorimotor differences between limbs that could become more apparent in studies with larger samples.

Dynamic balance assessment revealed a significant deficit only in the posteromedial reach direction of the Y-Balance Test, whereas no significant differences were observed in the anterior, posterolateral, or composite scores. Balance performance is influenced by multiple components, including lower extremity strength, range of motion, and coordination. In this context, the posteromedial direction has been associated with a greater contribution of multiple lower limb muscle groups compared with other directions, which may increase its sensitivity to detect subtle sensorimotor impairments (31, 32). Previous studies in individuals with anterior cruciate ligament (ACL) injury have reported that Y-Balance Test performance may not differ significantly from healthy controls, suggesting that athletes can adopt a variety of movement strategies to maintain task performance despite underlying impairments (33). This may help explain why no differences were observed in certain reach directions in the present study. Furthermore, variability in time since injury (3–6 months) may also have influenced dynamic balance performance, as athletes may differ in their level of neuromuscular recovery during this period. Despite the absence of between limb differences in the anterior and posterolateral directions, the observed moderate correlations between hop performance and Y-Balance Test scores indicate that dynamic balance remains closely related to functional lower extremity performance. Sport-specific characteristics in wrestlers, including high levels of neuromuscular conditioning and repeated exposure to complex movement patterns, may contribute to the preservation of balance performance in certain directions. Taken together, these findings suggest that while overall dynamic balance performance may be preserved through compensatory neuromuscular strategies in elite wrestlers, the posteromedial reach direction appears to be more sensitive in revealing subtle, injury-related deficits.

The single leg hop test is widely used as a functional indicator of lower extremity performance and neuromuscular control, particularly in athletes returning to sport after knee injury. Functional performance was significantly lower in the injured limb compared with the uninjured limb. Recent evidence has shown that athletes with a history of knee injury continue to demonstrate reduced hop performance, particularly during the return to sport period, with deficits of approximately 5%–10% compared with the contralateral limb (34). These findings suggest that reduced hop distance in the injured limb may reflect persistent deficits in strength, neuromuscular coordination, and dynamic knee stability despite the athletes having returned to training and competition. Current literature indicates that return to sport does not necessarily reflect full recovery of performance capacity, and residual neuromuscular deficits may remain in athletic populations following knee injury (35, 36).

In addition, hop test distance was also moderately correlated with Y-Balance Test reach distances, particularly in the posteromedial and posterolateral directions, indicating a potential relationship between dynamic balance and functional lower extremity performance in elite wrestlers. Hop test performance showed moderate correlations with subjective knee function (IKDC) and pain intensity (VAS), suggesting that athletes with better perceived knee function and lower pain levels tended to demonstrate superior functional performance. Together, these findings highlight the multidimensional nature of knee function, in which objective performance measures and subjective perceptions are closely related. This interpretation is supported by previous research showing that self-reported knee function may remain impaired following knee injury, even in athletes who have returned to sport (37).

Taken together, the findings of the present study suggest that athletes who have returned to full training after a knee injury may present subtle sensorimotor and functional asymmetries. Impairments in joint position sense at low flexion angles, deficits in dynamic balance in specific reach directions, and reduced hop performance indicate that proprioceptive accuracy and functional performance may not be fully restored despite continued training and competition. Furthermore, the observed relationships between hop performance, dynamic balance, and subjective knee function highlight the interconnected nature of neuromuscular control and functional capacity. These results emphasize the importance of incorporating comprehensive sensorimotor assessments, including joint position sense, dynamic balance, and hop tests, into return-to-sport evaluation protocols for wrestlers and other athletes with a history of knee injury.

### 4.1 Limitations of the study

This study has several limitations that should be considered when interpreting the findings. First, the relatively small sample size may limit the generalizability of the results, although the paired design enabled within-subject comparisons and reduced inter-individual variability. However, intra-rater or test–retest reliability was not evaluated within the present sample. Second, the cross-sectional design does not allow causal conclusions regarding the relationship between previous knee injury and the observed proprioceptive and functional differences. Third, the sample included athletes from both free-style and Greco-Roman wrestling styles as well as both male and female wrestlers, which may introduce variability related to sport-specific demands and physiological differences. In addition, proprioception was assessed using joint position sense at selected knee flexion angles, which may not fully represent proprioceptive performance during dynamic sport-specific tasks. Finally, different types of knee injuries were included in the analysis without subgroup stratification, and variability in time since injury (3–6 months) was not controlled across participants. These factors may have influenced the present findings and should be considered as potential confounding factors. Future studies with larger and more homogeneous samples, including muscle strength and neuromuscular control assessments as well as longitudinal follow-up evaluations, are warranted.

### 4.2 Strengths of the study

One of the main strengths of this study is that it was conducted in elite wrestlers competing at the national level, providing valuable insight into neuromuscular and functional characteristics in a high-performance athletic population. Evaluating athletes who have already returned to sport allows the identification of subtle residual deficits that may not be evident during routine clinical assessments. Another strength of the study is the within-subject comparison between injured and uninjured limbs in athletes with unilateral knee injury. This design reduces inter-individual variability and enables a more precise assessment of injury-related functional asymmetries. Additionally, the combined evaluation of proprioception, dynamic balance, and functional performance provides a comprehensive assessment of neuromuscular function in elite wrestlers.

## 5 Conclusions

Elite wrestlers with a history of unilateral knee injury demonstrated persistent deficits in proprioceptive accuracy at lower knee flexion angles, reduced posteromedial reach distance in the Y-Balance Test, and decreased single-leg hop performance. These findings indicate that subtle sensorimotor and functional asymmetries may persist despite return to sport. Incorporating proprioceptive and functional performance assessments into return to sport evaluations may help identify residual deficits and support targeted rehabilitation and injury-prevention strategies.

## Data Availability

The raw data supporting the conclusions of this article will be made available by the authors, without undue reservation.

## References

[1] Chaabene H Negra Y Bouguezzi R Mkaouer B Franchini E Julio U. Physical and physiological attributes of wrestlers: an update. J Strength Cond Res. (2017) 31(5):1411–42. 10.1519/JSC.0000000000001738 28030533

[2] Cieśliński I Gierczuk D Sadowski J. Identification of success factors in elite wrestlers-an exploratory study. PLoS One. (2021) 16(3):e0247565. 10.1371/journal.pone.0247565 33661963 PMC7932093

[3] James LP Haff GG Kelly VG Beckman EM. Towards a determination of the physiological characteristics distinguishing successful mixed martial arts athletes: a systematic review of combat sport literature. Sports Med. (2016) 46(10):1525–51. 10.1007/s40279-016-0493-1 26993133

[4] Chow AK Sigman ZW Pimental FJ Di Stefano MT Mazzocca AD. An epidemiological analysis of wrestling-related injuries: a 10-year analysis of national injury data. Orthop J Sports Med. (2026) 14(3):23259671261419521. 10.1177/23259671261419521 41798095 PMC12966515

[5] Powell JR Boltz AJ Robison HJ Morris SN Collins CL Chandran A. Epidemiology of injuries in National Collegiate Athletic Association Men's Wrestling: 2014–2015 through 2018–2019. J Athl Train. (2021) 56(7):727–33. 10.4085/1062-6050-429-20 34280284 PMC8293878

[6] Agel J Ransone J Dick R Oppliger R Marshall SW. Descriptive epidemiology of collegiate men’s wrestling injuries: National Collegiate Athletic Association Injury Surveillance System, 1988–1989 through 2003–2004. J Athl Train. (2007) 42(2):303–10. 17710180 PMC1941299

[7] Driban JB Hootman JM Sitler MR Harris KP Cattano NM. Is participation in certain sports associated with knee osteoarthritis? A systematic review. J Athl Train. (2017) 52(6):497–506. 10.4085/1062-6050-50.2.08 25574790 PMC5488840

[8] Ford K Schaver AL Leary S Keith JN Westermann RW. Return to sport after knee injuries in collegiate wrestling. Iowa Orthop J. (2023) 43(1):131–5. 37383862 PMC10296484

[9] Khalili-Borna D Honsik K. Wrestling and sports medicine. Curr Sports Med Rep. (2005) 4(3):144–9. 10.1097/01.csmr.0000306197.51994.16 15907266

[10] Bayati R Majelan AS Zarei H. Effects of the wrestling+injury prevention program in freestyle wrestlers: a two-arm randomized controlled trial. J Orthop Surg Res. (2025) 20(1):486. 10.1186/s13018-025-05911-z 40390025 PMC12087162

[11] Wroble RR Mysnyk MC Foster DT Albright JP. Patterns of knee injuries in wrestling: a six year study. Am J Sports Med. (1986) 14(1):55–66. 10.1177/036354658601400110) 3752347

[12] Ardern CL Taylor NF Feller JA Webster KE. Return-to-sport outcomes at 2 to 7 years after anterior cruciate ligament reconstruction surgery. Am J Sports Med. (2012) 40(1):41–8. 10.1177/0363546511422999 21946441

[13] Dingenen B Gokeler A. Optimization of the return-to-sport paradigm after anterior cruciate ligament reconstruction: a critical step back to move forward. Sports Med. (2017) 47(8):1487–500. 10.1007/s40279-017-0674-6 28078610

[14] Wang Z Chen N Cao S Gao L Geok SK Liu J. The effects of balance training on physical fitness and skill-related performance in basketball players: a systematic review. BMC Sports Sci Med Rehabil. (2025) 17(1):108. 10.1186/s13102-025-01164-9 40312426 PMC12044794

[15] Welling W Benjaminse A Lemmink K Gokeler A. Passing return to sports tests after ACL reconstruction is associated with greater likelihood for return to sport but fail to identify second injury risk. Knee. (2020) 27(3):949–57. 10.1016/j.knee.2020.03.007 32247810

[16] Noorbakhsh M Zarei M Hovanloo F Hoseini A Yaghoubitajani Z. Exploring the influence of a 10-week specific detraining on injury risk factors among elite young wrestlers: a prospective study. Sci Rep. (2025) 15(1):7348. 10.1038/s41598-025-91561-4 40025080 PMC11873154

[17] World Medical Association. World Medical Association Declaration of Helsinki: ethical principles for medical research involving human subjects. JAMA. (2013) 310(20):2191–4. 10.1001/jama.2013.281053 24141714

[18] Hawker GA Mian S Kendzerska T French M. Measures of adult pain: Visual Analog Scale for Pain (VAS Pain), Numeric Rating Scale for Pain (NRS Pain), McGill Pain Questionnaire (MPQ), Short-Form McGill Pain Questionnaire (SF-MPQ), Chronic Pain Grade Scale (CPGS), Short Form-36 Bodily Pain Scale (SF-36 BPS), and Measure of Intermittent and Constant Osteoarthritis Pain (ICOAP). Arthritis Care Res (Hoboken). (2011) 63(Suppl 11):S240–52. 10.1002/acr.20543 22588748

[19] Briggs KK Steadman JR Hay CJ Hines SL. Lysholm score and tegner activity level in individuals with normal knees. Am J Sports Med. (2009) 37(5):898–901. 10.1177/0363546508330149 19307332

[20] Irrgang JJ Anderson AF Boland AL Harner CD Kurosaka M Neyret P. Development and validation of the international knee documentation committee subjective knee form. Am J Sports Med. (2001) 29(5):600–13. 10.1177/03635465010290051301 11573919

[21] Relph N Herrington L Tyson S. The effects of ACL injury on knee proprioception: a meta-analysis. Physiotherapy. (2014) 100(3):187–95. 10.1016/j.physio.2013.11.002 24690442

[22] Shaffer SW Teyhen DS Lorenson CL Warren RL Koreerat CM Straseske CA. Y-balance test: a reliability study involving multiple raters. Mil Med. (2013) 178(11):1264–70. 10.7205/MILMED-D-13-00222 24183777

[23] Dingenen B Truijen J Bellemans J Gokeler A. Test-retest reliability and discriminative ability of forward, medial and rotational single-leg hop tests. Knee. (2019) 26(5):978–87. 10.1016/j.knee.2019.06.010 31431339

[24] Gokeler A Welling W Zaffagnini S Seil R Padua D. Development of a test battery to enhance safe return to sports after anterior cruciate ligament reconstruction. Knee Surg Sports Traumatol Arthrosc. (2017) 25(1):192–9. 10.1007/s00167-016-4246-3 27423208 PMC5315711

[25] Turck SM Faria Silva M Francys Vidmar M Goldani Ramos L Dias Zillmann H Mezzomo M. Using the single-leg hop test to determine knee extension strength after anterior cruciate ligament reconstruction. Orthop J Sports Med. (2025) 13(4):23259671241303520. 10.1177/23259671241303520 40336528 PMC12057934

[26] Cohen J. Statistical Power Analysis for the Behavioral Sciences. New York, NY, USA: Routledge (2013).

[27] Wellsandt E Failla MJ Snyder-Mackler L. Limb symmetry indexes can overestimate knee function after anterior cruciate ligament injury. J Orthop Sports Phys Ther. (2017) 47(5):334–8. 10.2519/jospt.2017.7285 28355978 PMC5483854

[28] Proske U Gandevia SC. The proprioceptive senses: their roles in signaling body shape, body position and movement, and muscle force. Physiol Rev. (2012) 92(4):1651–97. 10.1152/physrev.00048.2011 23073629

[29] Relph N Herrington L. The effects of ACL injury on knee proprioception: a meta-analysis. Knee. (2016) 23(2):144–53. 10.1016/j.knee.2015.10.010 24690442

[30] Lephart SM Pincivero DM Giraldo JL Fu FH. The role of proprioception in the management and rehabilitation of athletic injuries. Am J Sports Med. (1997) 25(1):130–7. 10.1177/036354659702500126 9006708

[31] Lee DK Kim GM Ha SM Oh JS. Correlation of the Y-balance test with lower-limb strength of adult women. J Phys Ther Sci. (2014) 26(5):641–3. 10.1589/jpts.26.641 24926122 PMC4047222

[32] Plisky PJ Rauh MJ Kaminski TW Underwood FB. Star excursion balance test as a predictor of lower extremity injury in high school basketball players. J Orthop Sports Phys Ther. (2009) 39(12):911–9. 10.2519/jospt.2009.3044 17193868

[33] Bulow A Bellemare A Anderson JE Leiter JRS MacDonald PB Peeler JD. Lower extremity kinematics of the Y-balance test in healthy and ACL injured adolescent females. Int J Sports Phys Ther. (2021) 16(2):381–92. 10.26603/001c.21529 33842034 PMC8016411

[34] Girdwood MA Crossley KM Rio EK Patterson BE Haberfield MJ Couch JL. Hop to it! A systematic review and longitudinal meta-analysis of hop performance after ACL reconstruction. Sports Med. (2025) 55(1):101–13. 10.1007/s40279-024-02121-1 39414723 PMC11787245

[35] Gill VS Tummala SV Han W Boddu SP Verhey JT Marks L. Athletes continue to show functional performance deficits at return to sport after anterior cruciate ligament reconstruction: a systematic review. Arthroscopy. (2024) 40(8):2309–2321.e2. 10.1016/j.arthro.2023.12.033 38220029

[36] Hsu CJ George SZ Chmielewski TL. Association of quadriceps strength and psychosocial factors with single-leg hop performance in patients with meniscectomy. Orthop J Sports Med. (2016) 4(12):2325967116676078. 10.1177/2325967116676078 28210647 PMC5298555

[37] Fältström A Hägglund M Hedevik H Kvist J. Self-reported knee function and activity level are reduced after primary or additional anterior cruciate ligament injury in female football players: a five-year follow-up study. Braz J Phys Ther. (2023) 27(6):100573. 10.1016/j.bjpt.2023.100573 38043159 PMC10703595

